# Effects of a nutrient mixture on immunohistochemical localization of cancer markers in human cervical cancer HeLa cell tumor xenografts in female nude mice

**DOI:** 10.3892/etm.2014.2127

**Published:** 2014-12-11

**Authors:** M.W. ROOMI, T. KALINOVSKY, J. CHA, N.W. ROOMI, A. NIEDZWIECKI, M. RATH

**Affiliations:** Dr Rath Research Institute, Santa Clara, CA 95050, USA

**Keywords:** HeLa, nutrient mixture, tumor growth, immunohistochemistry, Ki67, matrix metalloproteinase-2, matrix metalloproteinase-9, vascular endothelial growth factor, terminal deoxynucleotidyl transferase dUTP nick end labeling, B-cell lymphoma-2, cyclooxygenase 2, inducible nitric oxide synthase, glutathione S-transferase π

## Abstract

Although fully treatable in the early stages, once cervical cancer has metastasized, patient outcome is poor. The main objective of this study was to examine the effect of dietary supplementation with a nutrient mixture (NM) containing lysine, ascorbic acid, proline, green tea extract and other micronutrients on HeLa cell xenografts in nude female mice. Tumor growth was measured and immunohistochemical staining was evaluated for the following cancer markers: Ki67 (proliferation); matrix metalloproteinase (MMP)-2 and -9 (invasion/metastasis); vascular endothelial growth factor (VEGF) (angiogenesis); terminal deoxynucleotidyl transferase dUTP nick end labeling (TUNEL) and B-cell lymphoma 2 (Bcl-2) (apoptosis); cyclooxygenase 2 (COX-2) and inducible nitric oxide synthase (iNOS) (inflammation); and glutathione S-transferase π (GSTπ) (a general cancer marker). Following housing for a week, 5/6-week-old female athymic nude mice (n=12) were inoculated subcutaneously with 3×10^6^ HeLa cells in 0.2 ml phosphate-buffered saline and 0.1 ml Matrigel™ and randomly divided into two groups; control group mice were fed regular mouse chow and NM group mice the regular diet supplemented with 0.5% NM (w/w). After four weeks, the mice were sacrificed and their tumors were excised and processed for histology. The NM strongly inhibited the growth of HeLa xenografts in nude mice. The mean tumor weight was reduced to 59% (P=0.001) in the mice fed the NM compared with the tumor weight in the controlled diet mice. Ki67, MMP-2 and -9, VEGF, TUNEL, Bcl-2, COX-2, iNOS and GSTπ all showed a lower intensity and frequency of staining in the NM group compared with that in the control group. In conclusion, NM supplementation strongly inhibited tumor growth and cancer markers in female nude mice injected with HeLa xenografts.

## Introduction

Cervical cancer is the third most commonly diagnosed cancer and the fourth leading cause of female mortality worldwide, with >85% of the associated deaths occurring in developing countries (1). The American Cancer Society estimates ~12,360 new cases of invasive cervical cancer and ~4,020 mortalities in the United States for 2014 (2). The one-year survival rate for females with cervical cancer is 87% and the five-year survival rate is 68%. When detected early, the five-year survival rate for patients with invasive cervical cancer is 91% (3). Cervical cancer develops slowly, taking 10–15 years to develop into cancer from a pre-cancerous condition called dysplasia. Although fully treatable in the early stages, once the cancer has metastasized, patient outcome is poor.

Critical to tumor cell invasion are the processes of cell attachment, proteolytic degradation of the extracellular matrix (ECM) and migration through the disrupted matrix (4). Rath and Pauling (5) proposed that the use of nutrients, such as lysine and ascorbic acid, to target plasmin-mediated connective tissue degradation should be considered as a universal approach to control tumor growth and expansion. Binding to plasminogen active sites, lysine blocks plasminogen activation into plasmin by tissue plasminogen activator; thus, it modulates the plasmin-induced matrix metalloproteinase (MMP) activation cascade (6). We have previously developed strategies to inhibit cancer growth and its spread using complex micronutrient supplementation with select natural compounds, such as lysine, proline, ascorbic acid and green tea extract (7). This nutrient mixture (NM) demonstrated pleiotropic synergistic anticancer activity *in vivo* and *in vitro* in several cancer cell lines through the inhibition of cancer cell growth, MMP secretion, invasion, metastasis and angiogenesis (7).

In previous studies we found that NM significantly inhibited the proliferation of cervical cancer HeLa cells *in vitro*, the secretion of MMP-2 and -9, urokinase plasminogen activator activity and Matrigel™ invasion, and enhanced tissue inhibitor of matrix metalloproteinases 2 activity (8,9). In the present study the *in vivo* effects of NM supplementation on tumor growth and cancer markers in cervical cancer HeLa cell tumor xenografts in female nude mice were investigated. The cancer cell markers studied by tumor immunohistochemistry (IHC) were as follows: Ki67 (proliferation marker); MMP-2 and -9 (metastasis markers); vascular endothelial growth factor (VEGF) (angiogenesis marker); terminal deoxynucleotidyl transferase dUTP nick end labeling (TUNEL) and B-cell lymphoma 2 (Bcl-2) (apoptosis markers); cyclooxygenase-2 (COX-2) and inducible nitric oxide synthase (iNOS) (inflammatory markers) and glutathione S-transferase π (GSTπ) (specific cancer marker).

## Materials and methods

### Animals

Female athymic nude mice, approximately five weeks of age on arrival, were purchased from Simonsen Laboratories (Gilroy, CA, USA) and maintained in microisolator cages under pathogen-free conditions on a 12-h light/dark schedule for a week. All procedures were performed according to a protocol approved by an internal institutional Animal Safety Review Committee (Dr Rath Research Institute, Santa Clara, CA, USA) and followed guidelines for the humane and customary care and use of experimental animals.

### Experimental design

Following housing for a week, 5/6-week-old female athymic nude mice (n=12) were inoculated subcutaneously with 3×10^6^ HeLa cells in 0.2 ml phosphate-buffered saline and 0.1 ml Matrigel (BD Biosciences, Bedford, MA, USA). Following injection, the mice were randomly divided into two groups: the control group mice were fed regular Purina mouse chow (Laboratory Rodent Diet 5001; Purina Mills, Richmond, IN, USA) and the NM mice were fed the regular diet supplemented with 0.5% NM (w/w). During the study, the mice consumed, on average, 4 g/day of their respective diets; thus, the supplemented mice received ~20 mg NM/day. After four weeks, the mice were sacrificed and their tumors were excised, weighed and processed for histology. The mean weight of the mice at the beginning and end of the study did not differ significantly between the groups.

### Composition of the NM

The NM was composed of 700 mg vitamin C (as ascorbic acid and as Mg, Ca and palmitate ascorbate); 1,000 mg L-lysine; 750 mg L-proline; 500 mg L-arginine; 200 mg N-acetyl cysteine; 1,000 mg standardized green tea extract; 30 μg selenium; 2 mg copper; and 1 mg manganese. All nutrients were obtained from Vita-Tech International Inc. (Tustin, CA, USA). The certificate of analysis for the green tea extract obtained from US Pharma Lab. (North Brunswick, NJ, USA) indicated the following characteristics: Total polyphenol, 80%; catechins, 60%; epigallocatechin gallate, 35%; and caffeine 1.0%.

### IHC

Tumors were placed in a formalin cassette and sent to IDEXX (Sacramento, CA, USA) and HistoTox (Boulder, CO, USA) for analyses. Formalin-fixed samples of tumors were trimmed, processed, blocked, sectioned and stained with hematoxylin and eosin, and evaluated microscopically by IDEXX Pathology. The IHC of the tumor sections conducted by HistoTox Labs assessed Ki67, MMP-2 and MMP-9, VEGF, TUNEL, Bcl-2 and iNOS.

## Results

### Tumor growth

**Figure 1:**
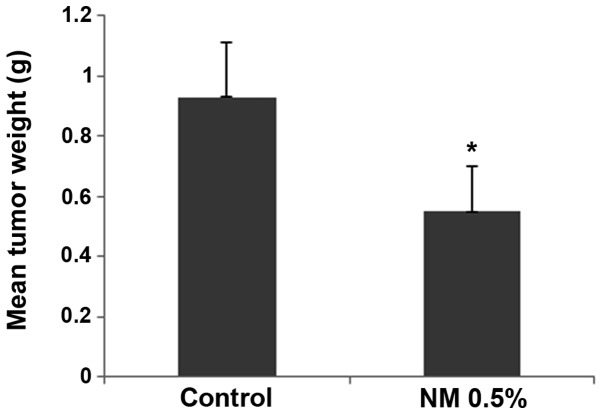
Effect of NM 0.5% supplementation on HeLa xenograft tumor weight. ^*^P=0.001 vs. the control. NM, nutrient mixture.

The NM strongly inhibited the growth of HeLa xenografts in female nude mice. The mean tumor weight was reduced to 59% (P=0.001) in mice with NM 0.5% dietary supplementation compared with the tumor weight in the controlled-diet mice, as shown in Fig. 1.

### Histology of tumors

**Figure 2:**
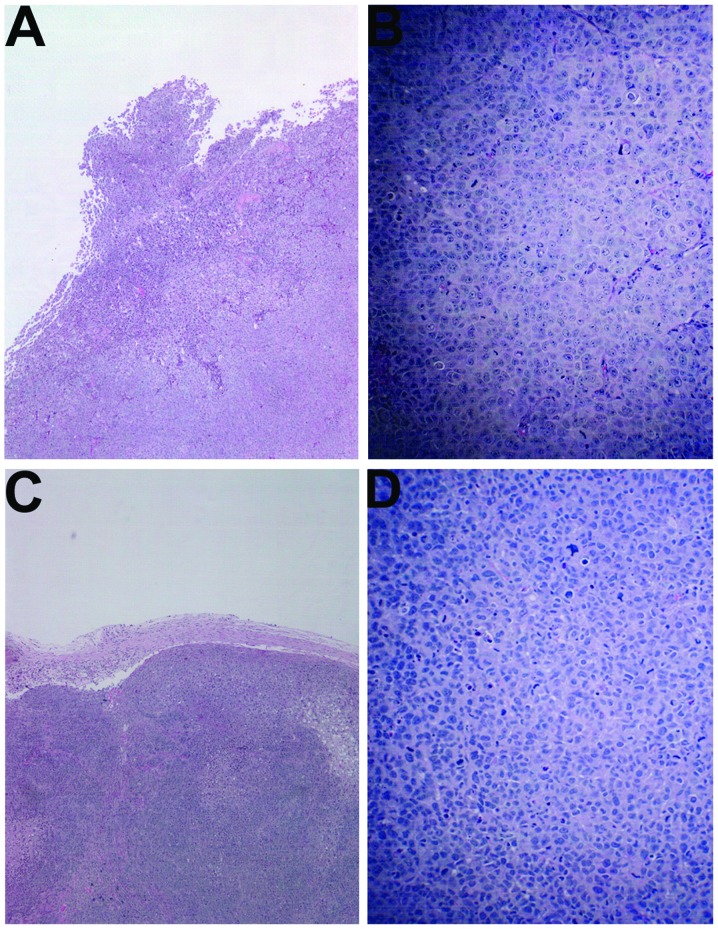
Histology of tumors in the control and NM groups (hematoxylin and eosin staining). (A) Control, magnification ×4; (B) control, magnification ×20 (C) NM, magnification ×4; (D) NM, magnification, ×20. NM, nutrient mixture.

The histology of the tumors in both groups was comparable, excepting a smaller tumor size in the NM-treated group. A salient feature of the results was that the tumor border in the untreated groups was ill defined, whereas in the NM group the fibrous capsule was prominent (Fig. 2).

### Proliferation: Ki67

**Figure 3:**
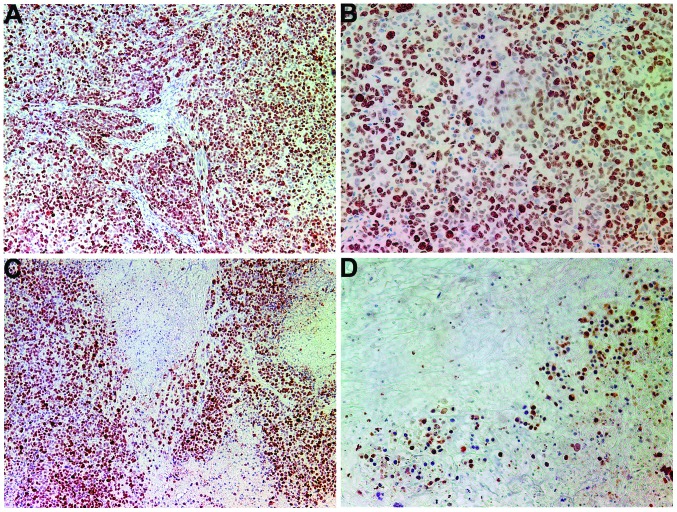
Effect of NM supplementation on Ki67 in representative tumors from the control and NM groups. (A) Control, magnification ×10; (B) control, magnification ×20; (C) NM, magnification ×10; (D) NM, magnification ×20. NM, nutrient mixture.

The proliferation marker Ki67 showed a lower intensity and frequency of staining in the NM group compared with that in the control group. Tumors from the control group showed ≥60% of cells positive for Ki67, with the positive cells dispersed uniformly throughout the tumor amid the Ki67-negative cells (Fig. 3A and B). The NM-treated tumor sections exhibited large areas of Ki67-negative nucleated cells amid areas of positive cells (Fig. 3C and D). The Ki67-positive cells were observed to aggregate circumferentially within the tumor capsule, away from the core, in the NM-treated tumors, whereas in the untreated tumors the Ki67-positive cells were found to be distributed uniformly throughout.

### Invasion/metastasis: MMP-2 and -9

#### MMP-2

**Figure 4:**
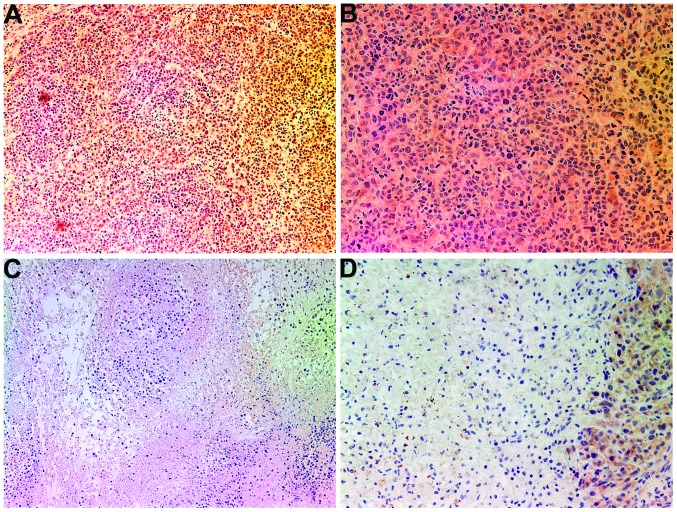
Effect of NM supplementation on matrix metalloproteinase-2 in representative tumors from the control and NM groups. (A) Control, magnification ×10; (B) control, magnification ×20; (C) NM, magnification ×10; (D) NM, magnification, ×20. NM, nutrient mixture.

Tumors from the control group showed intense uniform staining of MMP-2 in and around each cell (Fig. 4A and B), while the NM-treated tumors exhibited greatly reduced central expression and secretion of MMP-2 with a ring of peripheral cells positive for MMP-2 (Fig. 4C and D).

#### MMP-9

**Figure 5:**
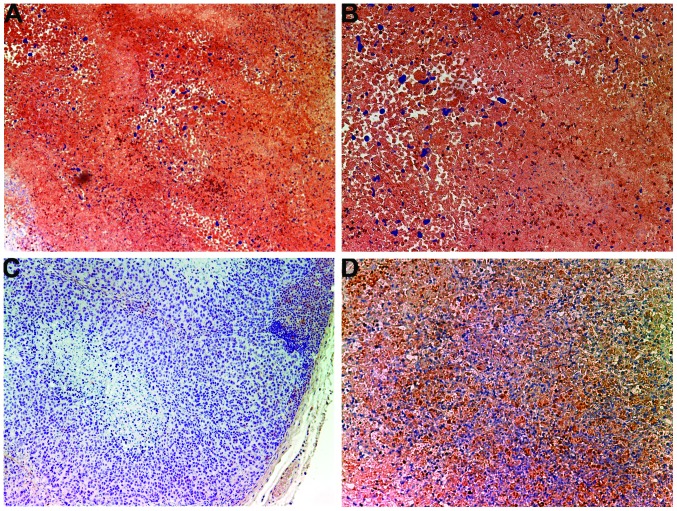
Effect of NM supplementation on matrix metalloproteinase-9 staining in representative tumors from the control and NM groups. (A) Control, magnification ×10; (B) control, magnification ×20; (C) NM, magnification ×10; (D) NM, magnification ×20. NM, nutrient mixture.

The NM-treated tumors showed less MMP-9 staining than did the control-group tumors. Untreated tumors exhibited large areas of MMP-9 (Fig. 5A and B), while NM-treated tumors had large areas of cells with no MMP-9 surrounding smaller populations of cells with abundant MMP-9 (Fig. 5C and D).

#### Angiogenesis: VEGF

**Figure 6:**
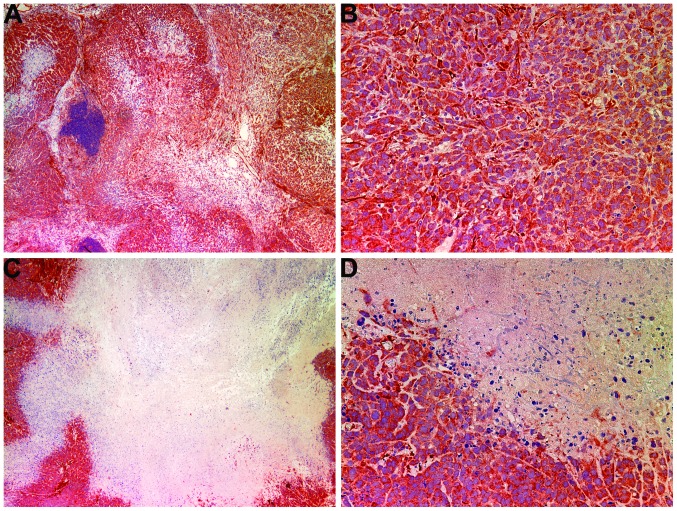
Effect of NM supplementation on vascular endothelial growth factor staining in representative tumors from the control and NM groups. (A) Control, magnification ×4; (B) control, magnification ×20; (C) NM, magnification ×4; (D) NM, magnification ×20. NM, nutrient mixture.

The majority of the cross-sectional areas of untreated tumors exhibited VEGF expression in a uniform distribution pattern (Fig. 6A and B). The NM-treated tumors showed markedly reduced VEGF staining, and large central areas of cells with no VEGF expression (Fig. 6C and D) were observed.

### Apoptosis: TUNEL and Bcl-2

#### TUNEL

**Figure 7:**
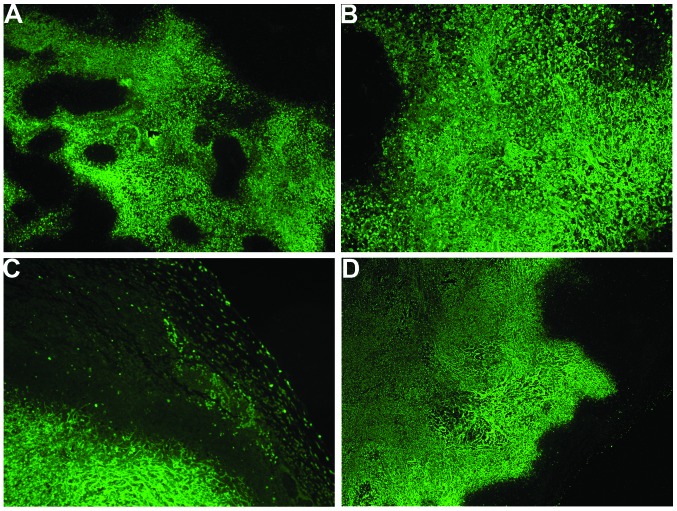
Effect of NM supplementation on terminal deoxynucleotidyl transferase dUTP nick end labeling staining in representative tumors from the control and NM groups. (A) Control, magnification ×4; (B) control, magnification ×10; (C) NM, magnification ×4; (D) NM, magnification ×4. NM, nutrient mixture.

Abundant apoptosis occurred in both treated and untreated tumors with more apoptosis occurring in the NM-treated group. The control group tumors showed regions of cells that were not undergoing apoptosis within larger regions of cells undergoing apoptosis for a heterogeneous pattern of normal interspersed with apoptotic cells (Fig. 7A and B). The NM-treated tumors had uniform areas of homogeneous apoptotic cells with peripheral areas of non-apoptotic cells (Fig. 7C and D). Some apoptosis was detected in the cells of the fibrous capsule of NM-treated tumors.

#### Bcl-2

**Figure 8:**
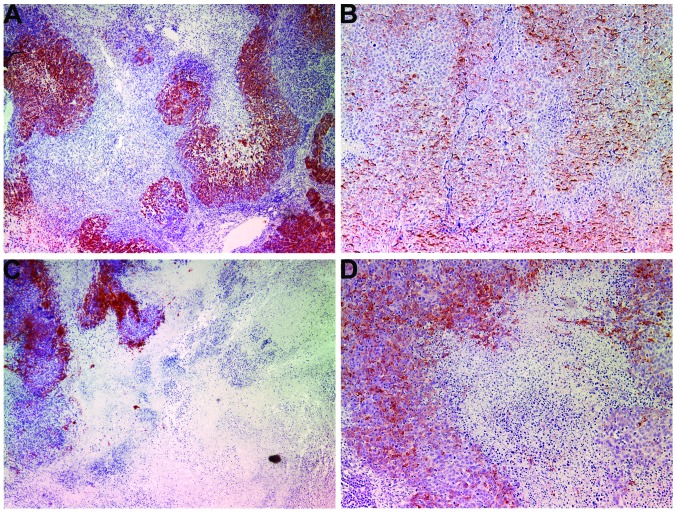
Effect of NM supplementation on B-cell lymphoma-2 staining in representative tumors from the control and NM groups. (A) Control, magnification ×4; (B) control, magnification ×10; (C) NM, magnification ×4; (D) NM, magnification ×10. NM, nutrient mixture.

High staining for Bcl-2, a pro-survival, anti-apoptotic protein, was observed in untreated tumors, whereas a reduced level of Bcl-2 was detected in the NM-treated tumors. The untreated tumors had extensive regions of Bcl-2-positive cells inside the tumors adjacent to and infiltrating regions of cells without Bcl-2 (Fig. 8A and B). The NM-treated tumors had vast regions of Bcl-2-free cells, which were clearly nucleated with considerably less Bcl-2 expression (Fig. 8C and D).

### Inflammation: COX-2 and iNOS

#### COX-2

**Figure 9:**
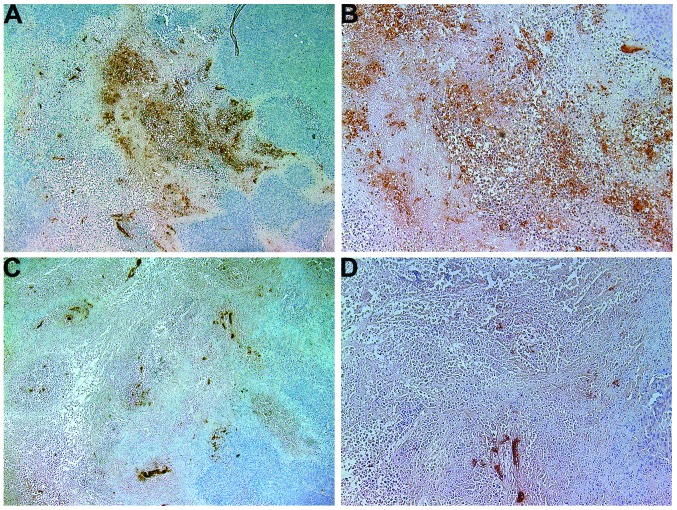
Effect of NM supplementation on cyclooxygenase-2 staining in representative tumors from the control and NM groups. (A) Control, magnification ×4; (B) control, magnification ×10; (C) NM, magnification ×4; (D) NM, magnification ×10. NM, nutrient mixture.

Tumors from the control group exhibited large dispersed regions of central COX-2 enzymes (Fig. 9A and B). The NM-treated tumors showed decreased levels of COX-2 (Fig. 9C and D).

#### iNOS

**Figure 10:**
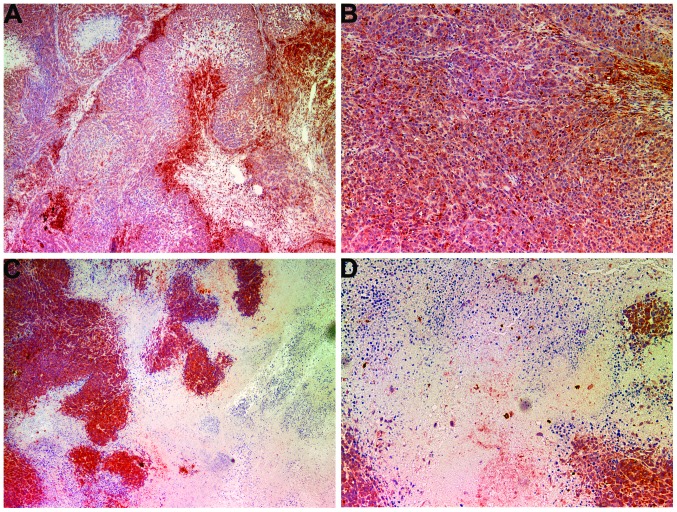
Effect of NM supplementation on inducible nitric oxide synthase staining in representative tumors from the control and NM groups. (A) Control, magnification ×4; (B) control, magnification ×10; (C) NM, magnification ×4; (D) NM, magnification ×10. NM, nutrient mixture.

Tumors from the control group had extensive diffuse cytoplasmic as well as intense punctate iNOS staining within the tumor, with the majority of the tumor positive for iNOS (Fig. 10A and B). By contrast, in the NM-treated group, tumors exhibited large areas devoid of iNOS (Fig. 10C and D).

#### Cancer marker GSTπ

**Figure 11:**
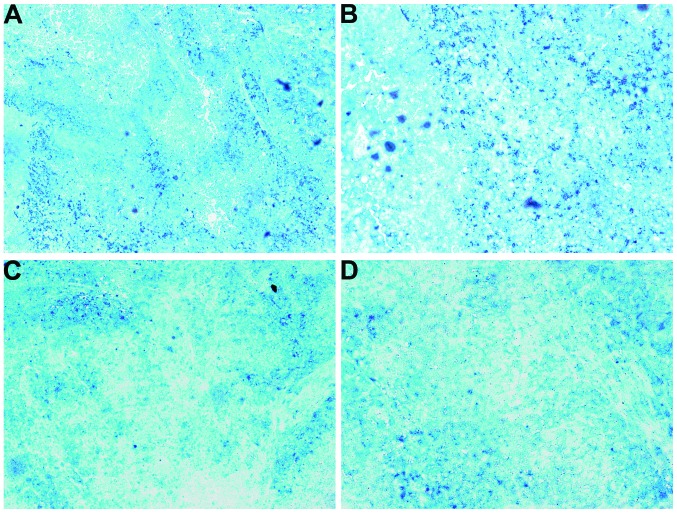
Effect of NM supplementation on glutathione S-transferase π staining in representative tumors from the control and NM groups. (A) Control, magnification ×10; (B) control, magnification ×20; (C) NM, magnification ×10; (D) NM, magnification ×20. NM, nutrient mixture.

The untreated tumors had homogeneous diffuse GSTπ expression as well as intense punctate staining (Fig. 11A and B). The NM-treated tumors exhibited a similar pattern but also with regions free of GSTπ (Fig. 11C and D).

## Discussion

In the present study, NM dietary supplementation of female nude mice challenged with HeLa xenografts resulted in a profound reduction in mean tumor weight compared with the control group. IHC staining of tumor markers confirmed this observation. The proliferation marker Ki67 was markedly reduced in the tumors from NM-supplemented mice. TUNEL staining showed abundant apoptosis in both tumor groups, with more apoptosis occurring in the NM-treated group. The control-group tumors showed regions of cells not undergoing apoptosis within larger regions of cells undergoing apoptosis. The NM-treated tumors showed uniform areas of apoptotic cells with peripheral areas of non-apoptotic cells.

The activity of MMPs, particularly MMP-2 and-9, on the degradation of the ECM plays a critical role in the formation of tumors and metastasis, and high MMP-9 levels have been found to correlate with the aggressiveness of cancers (10). Clinical studies have noted the association of MMP expression with the progression of cervical cancers (11,12). Bonfil *et al* (13,14) reported that tumor necrosis was an important source of gelatinase/type IV collagenase, mainly in its 92-kDa form, and thus played a major role in tumor invasion. In the present study, tumors from the control group showed intense uniform staining of MMP-2 in and around each cell, while tumors from the NM-supplemented mice exhibited greatly reduced central MMP-2 expression surrounded by a ring of peripheral cells positive for MMP-2. In addition, tumors from NM-supplemented mice showed less MMP-9 staining than did control-group tumors. Tumors from the control group exhibited large areas of MMP-9, whereas NM-treated tumors showed large areas of cells with no MMP-9 surrounding smaller areas with abundant MMP-9. In a previous *in vitro* study, cervical HeLa cells showed an intense band corresponding to MMP-2 and a faint band corresponding to MMP-9, which was enhanced with phorbol 12-myristate 13-acetate treatment. The NM completely blocked MMP-9 expression at 500 μg/ml and MMP-2 expression at 1,000 μg/ml (8).

The expression of the pro-angiogenic factor VEGF, which is critical for primary tumor growth and metastasis, was also found to be significantly lower in tumors from NM-supplemented mice than that in tumors from the control group. The majority of the cross-sectional areas of the control-group tumors exhibited VEGF expression in a uniform distribution pattern. The NM-treated tumors showed markedly reduced VEGF staining with large central areas of cells having no VEGF expression. In examining specimens immunohistochemically from normal cervical epithelium, carcinoma *in situ*, microinvasive carcinoma and invasive cervical squamous carcinoma (SCC), Fujiwaki *et al* (15) reported that VEGF expression progressively increased along a continuum from normal epithelium to invasive SCC (P<0.0001). Mathur *et al* (16) found serum VEGF-C upregulation to be a unique marker for the early diagnosis of cervical cancer metastasis.

In addition to promoting the progression of cancer, elevated pro-inflammatory cytokine levels have been associated with a variety of pathologies, such as fatigue, depression and cachexia (17,18). In studying the inflammatory markers COX-2 and iNOS in the present study, NM supplementation of the mouse diet was found to decrease COX-2 and iNOS expression. It was found that tumors from the NM-supplemented mice exhibited reduced COX-2 staining in comparison with tumors from the control group, which exhibited large dispersed regions of central COX-2 enzymes. Furthermore, tumors from the control group had extensive diffuse cytoplasmic as well as intense punctate iNOS staining within the tumor, with the majority of the tumor positive for iNOS. By contrast, in the NM-treated group, tumors exhibited large areas devoid of iNOS. In previous studies, the NM has been shown to have an inhibitory effect on inflammatory mediators, such as COX-2, in various cancer cell lines (7,19,20).

Nuclear GSTπ is important in the diagnosis of cancer as it is expressed abundantly in tumor cells (21). This enzyme plays a role in the detoxification of both endogenous and exogenous electrophiles that can react with cellular components such as DNA. In the present study, tumors from the control group exhibited diffuse, uniform GSTπ expression, as well as intense punctate staining. Tumors from the NM-treated mice exhibited a similar pattern, but with further regions free of GSTπ.

Rath and Pauling (5) proposed that nutrients such as lysine and ascorbic acid could potentially modulate tumor growth and expansion by acting as natural inhibitors of ECM degradation, inhibiting MMP activity and strengthening the integrity of the connective tissue surrounding cancer cells. Based on this approach, a complex of nutrients was developed to affect multiple key cancer mechanisms at once through their synergistic effects. The roles of the individual components of this mixture in relation to the critical aspects of cancer are described below. Optimal ECM structure depends upon adequate supplies of ascorbic acid, lysine and proline to maintain the synthesis and hydroxylation of collagen fibers. Furthermore, lysine contributes to the stability of the ECM as a natural inhibitor of plasmin-induced proteolysis (5,22). Manganese and copper are essential cofactors for collagen formation, and the potency of green tea extract in modulating certain processes associated with cancer progression, including cancer cell growth, metastasis and, angiogenesis, is well documented (23–27). N-acetyl cysteine and selenium have been demonstrated to inhibit tumor cell MMP-9 and invasive activities, as well as the migration of endothelial cells through the ECM (28–30). Ascorbic acid demonstrates cytotoxic and antimetastatic actions on malignant cell lines (31–36) and patients with cancer have been found to have low levels of ascorbic acid (37,38). Low levels of arginine, a precursor of nitric oxide (NO), can limit the production of NO, which has been demonstrated to mainly act as an inducer of apoptosis (39).

The results presented in this study showed the potent NM-induced inhibition of tumor growth and cancer markers in female nude mice injected with HeLa xenografts, suggesting the therapeutic value of this specific nutrient complex in the treatment of cervical cancer. Supplementation with the NM has beneficial effects in modulating cancer markers of proliferation (Ki67), invasion/metastasis (MMP-2 and -9), angiogenesis (VEGF), apoptosis (TUNEL and Bcl-2) and inflammation (COX-2 and iNOS), as well as the general cancer marker GSTπ. Furthermore, in contrast to the toxic side effects of current treatments, the micronutrient mixture has been shown to be a safe therapeutic agent. In a previous *in vivo* study addressing safety issues, we found that gavaging adult female Osteogenic Disorder Shionogi rats (weighing 250–300 g) with NM (at 30, 90 or 150 mg/day for seven days) resulted in neither adverse effects on vital organs (heart, liver and kidney) nor on the associated functional serum enzymes, indicating the safety of this mixture even at these high doses, which far exceed the normal equivalent dosage of the nutrient (40).
